# Pulsed‐Electrical Field Ablation Completes Pulsed‐Field Ablation—Lessons Learned on Tissue Proximity Indication in Pulmonary Vein Isolation

**DOI:** 10.1111/jce.70026

**Published:** 2025-07-25

**Authors:** Malte Kranert, Christian Scheckenbach, Tobias Harm, Meinrad Gawaz, David Heinzmann

**Affiliations:** ^1^ Department of Cardiology and Angiology University Hospital of Tuebingen Tuebingen Germany; ^2^ Department of Pediatric Cardiology, Pulmonology and Intensive Care Medicine University Children's Hospital Tuebingen Germany

**Keywords:** atrial fibrillation, catheter ablation, pulsed‐field ablation, tissue proximity indication

## Abstract

**Introduction:**

Pulmonary vein isolation (PVI) in atrial fibrillation (AF) is the current cornerstone for AF ablation. Besides thermal ablation techniques, nonthermal pulsed‐field ablation (PFA) is rapidly transforming the way PVI is achieved. As every new technology has its own learning curve, we would like to report the lessons we learned by treating a 63‐year‐old woman admitted for paroxysmal AF ablation, with symptomatic palpitations.

**Methods and Results:**

We initially performed PVI with a pulsed‐electrical field variable‐loop circular catheter with the CARTO 3 mapping system. When the patient experienced recurrence of AF even after 3 months, the scheduled reablation procedure showed a reconnection of both posterior carinae. When revisiting the initial map, both recurrences occurred at the points where tissue proximity indication (TPI) during ablation was likely not sufficient, leading to reversible electroporation and subsequent reconnection of the veins. Those remaining areas were again ablated using PFA energy with a linear ablation catheter.

**Conclusion:**

TPI‐based contact filtering for transmural lesion success and durability seems to be a relevant tool to ensure irreversible electroporation and is likely of prognostic relevance.

## Graphical abstract

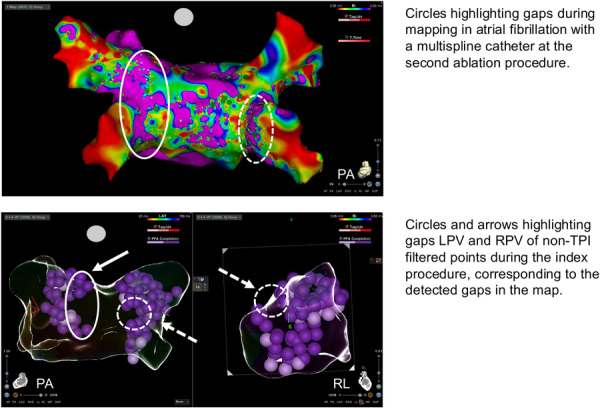

**Abbreviations**
AF: atrial fibrillation
AP: anterior posterior projection
LAO: left anterior oblique
LPV: left pulmonary veins
PA: posterior anterior projection
PFA: pulsed‐field ablation
PV: pulmonary vein
PVI: pulmonary vein isolation
RL: right lateral
RPV: right pulmonary veins
TPI: tissue proximity indication
VLCC: variable‐loop circular catheter

## 1 Introduction

Pulmonary vein isolation (PVI) in the treatment of atrial fibrillation (AF) remains the standard treatment in symptomatic AF [1]. While the development of different pulsed‐field ablation (PFA) catheter designs and voltage setups is ongoing, freedom from arrhythmia is still regarded to be equal to cryoballon ablation or radiofrequency ablation in PVI [2, 3]. In 2024, a variable‐loop circular catheter (VLCC) design integrated into a 3D mapping system (CARTO 3) was released to the electrophysiological community [4]. Safety and efficacy were shown to be equal compared to radiofrequency ablation [5, 6]. As every new technology and every new catheter design has its own learning curve, we would like to report the following case.

## 2 Case Report

A 63‐year‐old woman was referred to our tertiary center for PVI due to ongoing symptomatic paroxysmal AF. Conservative treatment with the maximum tolerable dose of oral beta‐blocker did not relieve the symptoms so a rhythm control strategy was planned. The patient additionally received antihypertensive therapy, showed normal left ventricular function, and coronary artery disease had been ruled out in the past.

**Figure 1:**
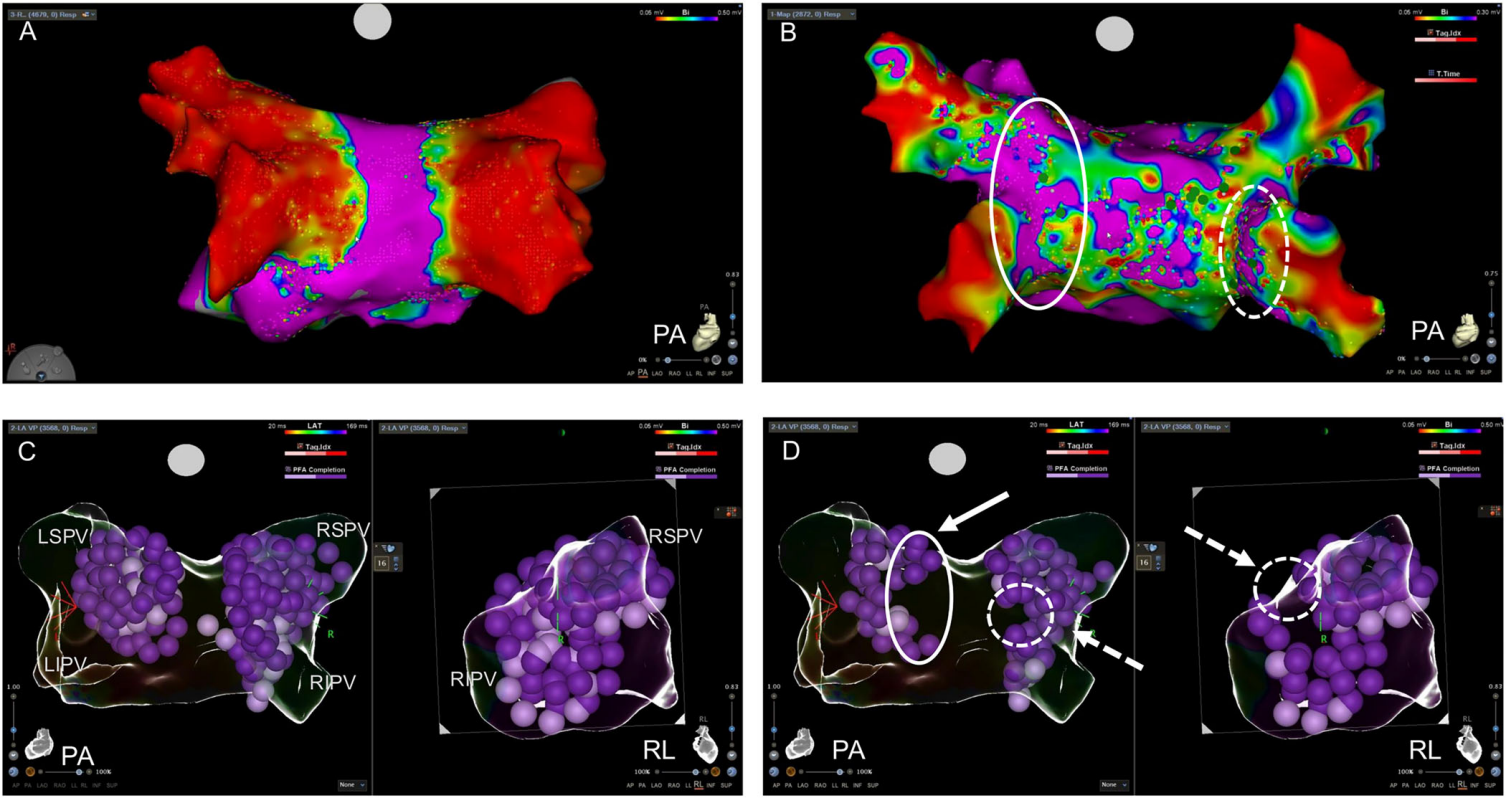
(A) Left atrium map postablation using a high‐resolution map with a multispline catheter (OCTARAY), posterior view. (B) The left atrium in the redo procedure, mapped in atrial fibrillation with a multispline catheter (PENTARAY), posterior view, white circles indicating reconnection sites. (C) Ablation point tags of the index procedure, non‐TPI filtered, showing PA and RL, (D) ablation point tags of the index procedure, TPI‐filtered, highlighting the gaps at the left and right pulmonary veins of non‐TPI tags with a white arrows and circles (continuous lines: LPV, dashed lines: RPV).

PVI was achieved using the VLCC in combination with a 3D multispline mapping catheter and the CARTO 3 mapping system with a pre‐ and postablation map. Figure 1A shows the initial map during the index procedure postablation with the multispline mapping catheter (OCTARAY). The patient was in the initial cohort of patients treated with the new ablation catheter representing the learning curve phase of the first 10 patients treated in our center. No low‐voltage areas < 0.5 mV were detected outside the pulmonary veins (PVs). A total of 63 energy deliveries consisting of 19 cycles with 3 applications each were applied, 5 cycles were stopped before completion. Additional ablations were performed at the right PVs at the carina and at the LSPV. After procedural success and confirming entrance and exit block, recurrence of AF occurred 1 month after the index procedure, with durations of up to 24 h. After the third recurrence, antiarrhythmic medication with amiodarone was advised, but ongoing therapy was stopped by the patient due to gastrointestinal side effects. Thus, a reablation was scheduled using a pentaspline mapping catheter and an irrigated tip, contact force sensing catheter. The voltage map was acquired under AF, as several periprocedural electrical cardioversions failed to establish a stable sinus rhythm (Figure 1B). Reconnections at both posterior carinae, especially of the left PVs, were detected. Additionally, several low‐voltage areas outside the PVs were detected under AF (low‐voltage area < 0.5 mV, TPI filtered points for map acquisition). When revisiting the initial map, both reconnections occurred at points where tissue proximity indication (TPI) during ablation was likely not sufficient, leading to reversible electroporation and subsequent reconnection of the veins (Figure 1C,D).

**Figure 2:**
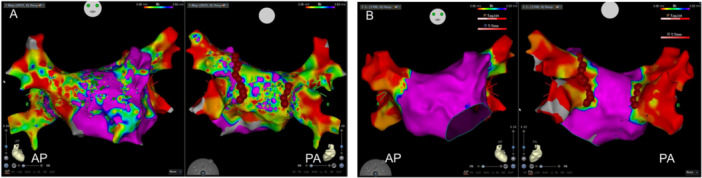
(A) The left atrium in the redo procedure, mapped in atrial fibrillation, TPI‐filtered point acquisition, low‐voltage area considered in signals < 0.05 mV, with a multispline catheter (PENTARAY), anterior view, posterior view, and ablation tags, (B) the left atrium in the redo procedure, postablation, TPI‐filtered point acquisition, low‐voltage area considered in signals < 0.05 mV, mapped in paced rhythm with a multispline catheter (PENTARAY), anterior view, posterior view, and ablation tags.

Reisolation of the PVs was achieved using PFA energy (Centauri, CardioFocus) applied over the irrigated tip, contact force sensing catheter to avoid thermal esophageal damage. Reablation was completed with 10 consecutive ablations for the left PVs, and 4 ablations for the right PVs (Figure 2A). Entrance and exit block of all PVs was confirmed. Electrical cardioversion was performed and the remap of the left atrium in paced rhythm did not reveal any low‐voltage zones outside the PVs and showed narrow border zones with no relevant tissue damage outside of the ablation areas (Figure 2B). High‐rate atrial burst stimulation did not induce any atrial tachycardia.

The patient was discharged the next day and clinical short‐term follow‐up of 1 month did not reveal a recurrence of AF.

## 3 Discussion

With new technology becoming widely available, physicians face the responsibility to build their skills with those new tools and to be mindful of procedural challenges that could limit the patients benefit.

This case was a great learning opportunity for our team leveraging all available data and tools to increase ablation results. After insufficient irreversible PFA, short‐term electric stunning can mimic electric isolation when performing PVI [2]. One of many factors in achieving a permanent lesion is TPI. As previously shown in animal models, insufficient tissue proximity during PFA delivery results in nondurable or nontransmural lesions in ~50%, while transmural lesions were performed in all applications with adequate tissue proximity, as assessed by the TPI [7]. When looking at clinical results, Saito and colleagues highlighted the increase of acute PVI failure when TPI showed no contact during energy delivery, as observed by Matsuura et al. [8, 9]. Similar to our case, TPI‐positive applications were less likely at the posterior carina of the left PVs [8]. This could be due to less contact, when the VLCC has more push to the anterior carina, thus not reaching adequate contact at the posterior carina. Another challenge in this case could be the small atrium, leading to difficult ostial catheter placement, especially in the small left PVs. Additionally, Saito and colleagues observed that bipolar voltage > 2.2 mV resulted in acute PV gap at the index procedure, interestingly not reflected by atrial‐wall thickness > 2 mm, assessed via computer tomography [8]. In the initial inspIRE trial, splitted in two phases, first‐pass PVI was reported in 96% and 97.1%, respectively [4]. In the follow‐up, when reablation procedures were performed, reconnections of ~72.5% of the veins (37/58) were detected [6]. In the following AdmIRE trial, in patients representing a repeat ablation procedure (*n* = 23; 9%), 51/91 (56%) of the veins were reconnected [5]. At the time point of the initial trials, TPI‐positive ablation lesions were not mandatory, a total number of 48 ablations, 12 ablations per vein, were regarded as sufficient for PVI [4, 6]. Due to incomplete energy delivery at the RIPV there were performed six completed ablations per RPV, to address the carina region adequately. To sum up, this case highlights the importance of accurate catheter positioning to ensure powerful TPI‐positive lesions with great tissue penetration. Filtering energy applications for TPI‐positive points after the first set of ablations and ensuring that the catheter is in positions with good TPI for the ongoing sets of ablations is likely able to reduce inefficient ablation lesions, avoiding overdosing of repeated ineffective ablations and thus safety and durability [7]. These considerations are also reflected in the step‐by‐step guide of Nair and colleagues, which highlights practical issues in the real‐world application [10]. Comparing the circular VLCC with other PFA catheter designs is difficult, as different voltages, pulse designs, and the directionality of energy delivery are unique to each tool and result in different challenges regarding handling and system integration. The great advantage of the VLCC is the seamless integration in the 3D‐mapping environment, reducing fluoroscopy time and providing voltage information beyond the PVs, whereas mapping integration for other PFA catheters will likely be widely available later this year.

## 4 Conclusion

This case highlights the importance of accurate lesion placement and TPI filtering when assessing ablation lesions to ensure durability with the current generation VLCC. Using all technological resources to improve patient outcomes is important to overcome anatomical and physiological limitations in this exciting new era of electrophysiology.

## Data Availability

The authors have nothing to report.
